# Web questionnaire survey of physicians and patients on the side effects of trifluridine/tipiracil

**DOI:** 10.1038/s41598-026-50912-5

**Published:** 2026-05-22

**Authors:** Takako Eguchi Nakajima, Ryota Okude, Takuma Tone, Ayako Oshika, Ayako Yokomizo

**Affiliations:** 1 Department of Early Clinical Development, Kyoto University Graduate School of Medicine, 54 Kawaharacho, Shogoin, Sakyo-Ku, Kyoto, 606-8507 Japan; 2 Medical Affairs Department, Taiho Pharmaceutical Co., Ltd, Tokyo, Japan

**Keywords:** Trifluridine/tipiracil, Supportive care, Non-hematologic toxicities, Colorectal cancer, Gastric cancer, Cancer, Gastroenterology, Health care, Medical research, Oncology

## Abstract

Trifluridine/tipiracil (FTD/TPI) is effective for advanced colorectal and gastric cancers; however, limited real-world data exist on physician and patient experiences with non-hematologic toxicities such as nausea, anorexia, and fatigue. We investigated the real-world incidence and management of these side effects from both perspectives. A web-based questionnaire survey was conducted among physicians experienced in prescribing FTD/TPI and patients with colorectal or gastric cancer who were receiving or had received these medications. Patients reported non-hematologic toxicities‒fatigue, anorexia, nausea, diarrhea, and vomiting‒more frequently and earlier than physicians anticipated. Fatigue and anorexia were the most distressing side effects for patients and the most challenging for physicians to manage. Discrepancies existed between physicians and patients regarding communication and side-effect management strategies. Although nausea and vomiting were manageable with antiemetics, clear management methods for fatigue and anorexia have not been established. Patients valued detailed explanations about medication administration, non-hematologic side effects, and costs more than physicians recognized. In FTD/TPI therapy, a discrepancy exists between physicians’ and patients’ perceptions of non-hematologic toxicities, which may pose challenges in effective symptom management. Symptoms such as fatigue and anorexia appear to be less adequately addressed, highlighting the need for continued efforts to improve management strategies.

**Supplementary Information:**

The online version contains supplementary material available at 10.1038/s41598-026-50912-5.

## Introduction

Trifluridine/tipiracil (FTD/TPI, Lonsurf) combination tablets comprise trifluridine and tipiracil hydrochloride in a molar ratio of 1:0.5. Trifluridine is absorbed by cancer cells, where it is phosphorylated by thymidine kinase and subsequently incorporated into deoxyribonucleic acid (DNA), thereby causing DNA dysfunction and exerting antitumor activity. Typically, orally administered trifluridine is degraded by thymidine phosphorylase (TP); however, tipiracil hydrochloride inhibits TP, thereby increasing systemic exposure to trifluridine and enhancing its therapeutic efficacy^1^.

FTD/TPI has demonstrated a survival benefit in phase III clinical trials for both advanced colorectal and gastric cancers. FTD/TPI improved overall survival compared to placebo in the RECOURSE trial for colorectal cancer (hazard ratio [HR] 0.68)^2^. The SUNLIGHT trial showed further survival benefit when FTD/TPI was combined with bevacizumab compared to FTD/TPI alone (HR 0.61)^3^. In gastric cancer, the TAGS trial also demonstrated a significant survival advantage for FTD/TPI over placebo (HR 0.69)^4^. Based on these findings, FTD/TPI has been approved and recommended as a standard third-line or later treatment for advanced colorectal and gastric cancers.

Myelosuppression, particularly neutropenia, is a prominent adverse event associated with FTD/TPI. In the RECOURSE trial, neutropenia occurred in 67% of patients (Grade ≥ 3 in 38%), and in the TAGS trial, in 53% (Grade ≥ 3 in 34%). Gastrointestinal toxicities and fatigue were also frequently reported. In RECOURSE, the incidence of nausea was 48%, vomiting 28%, anorexia 39%, and fatigue 35%. In the TAGS trial, nausea was reported in 37%, vomiting in 25%, anorexia in 34%, and fatigue in 27% of patients.

Based on the antiemetic guidelines established by the Japanese Society of Clinical Oncology, oral fluoropyrimidine agents (including S-1, UFT, and capecitabine) are associated with a low emetogenic risk, whereas FTD/TPI is categorized as having a moderate risk^5^. According to the MASCC/ESMO guidelines, the emetogenic risk of FTD/TPI is considered low or minimal; however, the ASCO guidelines classify it as moderate or high^6,7^. Surveys conducted in outpatient treatment settings have identified nausea and vomiting as the second most distressing symptoms among male patients with gastrointestinal cancer and the fifth most distressing among female patients, underscoring the critical importance of managing these symptoms during treatment^8^.

In addition to nausea and vomiting, other non-hematologic toxicities such as fatigue, anorexia, and diarrhea are frequently observed with FTD/TPI treatment; however, their real-world management remains poorly characterized. Data from 467 patients receiving anticancer treatment at Memorial Sloan Kettering Cancer Center revealed that healthcare professionals tend to underreport symptoms, such as nausea, appetite loss, and fatigue, compared to patient-reported outcomes, highlighting a perceptual discrepancy between patients and medical staff^9^. Moreover, the Toxicity over Time (ToxT) analysis has shown that conventional reporting may overlook the timing and persistence of adverse events, particularly with oral therapies, highlighting the need for longitudinal monitoring to fully capture their impact on patients’ quality of life^10^.

In this study, we conducted a web-based questionnaire survey of both physicians and patients to characterize and compare their perceptions regarding the incidence, timing, and management of non-hematologic toxicities associated with trifluridine/tipiracil (FTD/TPI) in real-world clinical practice. The primary objective was to identify potential gaps in perception between physicians and patients, particularly for symptoms that are difficult to evaluate objectively, such as fatigue and anorexia.

Given the descriptive nature of this survey, no predefined confirmatory hypotheses were established, and all analyses were exploratory. The findings of this study are expected to provide practical insights into unmet needs in symptom monitoring, patient education, and supportive care, and to inform strategies for improving communication and multidisciplinary management in routine oncology practice.

## Results

**Fig. 1:**
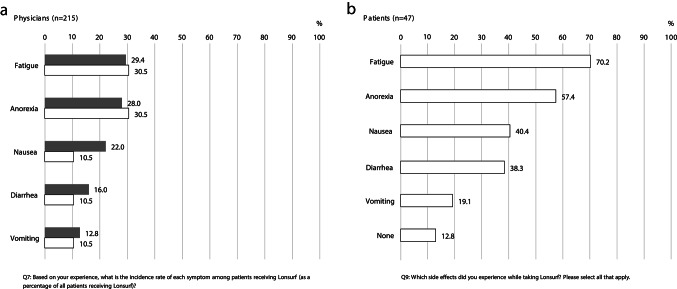
Proportion of non-hematologic toxicity. (**a**) Proportion of patients receiving FTD/TPI therapy for whom physicians anticipated the occurrence of non-hematologic toxicities. Black bars: mean values; white bars: median values – Questionnaire item Q7. (**b**) Proportion of patients who experienced non-hematologic toxicities during FTD/TPI therapy – Questionnaire item Q9.

Between January 29 and May 31, 2024, data were collected from 215 physicians and 47 patients. The background characteristics of physicians and patients are summarized in Tables 1 and 2. In the physician survey, the anticipated incidence proportion of non-hematologic toxicities were as follows: fatigue (29.4%), anorexia (28.0%), nausea (22.0%), diarrhea (16.0%), and vomiting (12.8%) in mean values (Fig. 1a). In contrast, the patient survey reported proportion of fatigue (70.2%), anorexia (57.4%), nausea (40.4%), diarrhea (38.3%), and vomiting (19.1%) (Fig. 1b). Notably, patient-reported proportion exceeded the expectations of physicians across all categories.

**Table 1:** Characteristics of participating physicians (n = 215).

		n	%
Clinical Department	Gastroenterology	63	29.3
	Oncology	32	14.9
	General Surgery	12	5.6
	Gastrointestinal Surgery	108	50.2
Number of Beds	100‒199	20	9.3
	200‒299	14	6.5
	300‒399	41	19.1
	400‒499	36	16.7
	> 500	104	48.4
Type of Facility	University Hospital (National/Public University Hospital, Private University Hospital)	48	22.3
	National/Public Hospital (Cancer Center, Cancer Specialty Hospital)	20	9.3
	National/Public Hospital (Other than the above)	60	27.9
	General Hospital (other than the above)	87	40.5
Certified Physician / Society Membership, etc	Diplomate, Subspecialty Board of Medical Oncology, (certified by the Japanese Society of Medical Oncology [JSMO])	51	23.7
	Certified cancer treatment physician (certified by the Japanese Board of Cancer Therapy [JBCT])	125	58.1
	Member of JSMO	84	39.1
	Member of the Japan Society of Clinical Oncology (JSCO)	96	44.7
	Main facility is a designated cancer care hospital	115	53.5
	None of the above	20	9.3
Treatment schedule	Standard regimen (5 days on, 2 days off × 2, 14 days off, q4w)	138	64.2
	First cycle started with the q4w regimen but switched to the q2w regimen due to AEs	54	25.1
	5 days on, 9 days off, q2w	23	10.7
		Median	Range
No. of patients Prescribing FTD/TPI per year	Gastric cancer FTD/TPI monotherapy	2	0‒25
	Gastric cancer FTD/TPI + ramucirumab	0	0‒15
	Colorectal cancer FTD/TPI monotherapy	2	0‒35
	Colorectal cancer FTD/TPI + bevacizumab	3	0‒25

FTD/TPI, trifluridine/tipiracil; q4w, every 4 weeks; q2w, every 2 weeks; AE, adverse event.

**Table 2:** Characteristics of participating patients (n = 47).

		n	%
Cancer Type	Gastric cancer	11	23.4
	Colorectal cancer	36	76.6
Sex	Male	27	57.4
	Female	20	42.6
Age	< 60 years	16	34.0
	≥ 60 years	31	66.0
Medication status	Currently taking FTD/TPI (not discontinued)	45	95.7
	Discontinued medication (< 3 months since the final dose)	2	4.3
	Discontinued medication (≥ 3 months since the final dose)	0	0.0
Treatment duration	< 1 month	10	21.3
	≥ 1 month and < 6 months	22	46.8
	≥ 6 months	15	31.9
Treatment schedule	Standard regimen (5 days on, 2 days off × 2, 14 days off, q4w)	31	66.0
	5 days on, 9 days off, q2w	10	21.3
	First cycle started with the q4w regimen but switched to the q2w regimen	4	8.5
	Others	2	4.3

FTD/TPI, trifluridine/tipiracil; q4w, every 4 weeks; q2w, every 2 weeks; AE, adverse event.

**Fig. 2:**
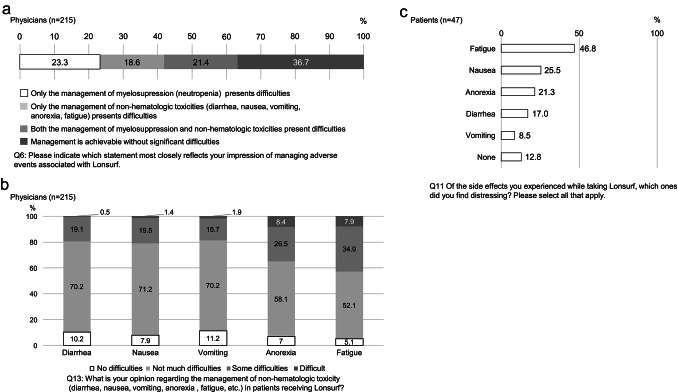
Impressions of side effects of FTD/TPI. (**a**) Physicians’ impressions of side-effect management – Questionnaire item Q6. (**b**) Physicians’ impressions of the management of each non-hematologic toxicity (diarrhea, nausea, vomiting, anorexia, fatigue) – Questionnaire item Q13. (**c**) Severe side effects experienced by patients (multiple responses allowed) – Questionnaire item Q11.

Patients generally experienced side effects earlier than physicians had anticipated (Supplementary Fig. S1). Among physicians, 40.0% reported difficulties in managing non-hematologic toxicities (Fig. 2a), with particularly high proportions indicating that they were “difficult” or “some difficulties” by fatigue (42.8%) and anorexia (34.9%) (Fig. 2b). The degree of management difficulty also varied by medical specialty; among medical oncologists, a higher proportion reported being able to manage these toxicities without considerable struggle (56.3%) (Supplementary Fig. S2).

Fatigue was the most prevalent distressing symptom reported by patients (46.8%), followed by nausea (25.5%) and anorexia (21.3%) (Fig. 2c). Fatigue was also the side effect most frequently selected as the single most distressing one (27.7%; data not shown). According to responses to the questionnaire adapted from the patient-reported outcomes version of the Common Terminology Criteria for Adverse Events (PRO-CTCAE)^11^, patients who experienced fatigue and anorexia most commonly rated the severity as "moderate," reported by 54.5% and 51.9%, respectively, with 39.4% and 37.0% indicating that these symptoms " interfered with my daily activities to some extent". In contrast, nausea was rated as more severe than fatigue or anorexia, with 31.6% of patients describing it as "severe." Regarding frequency, 26.3% of patients reported experiencing nausea "almost always," whereas 47.4% described it as “frequent” (Supplementary Fig. S3).

**Fig. 3:**
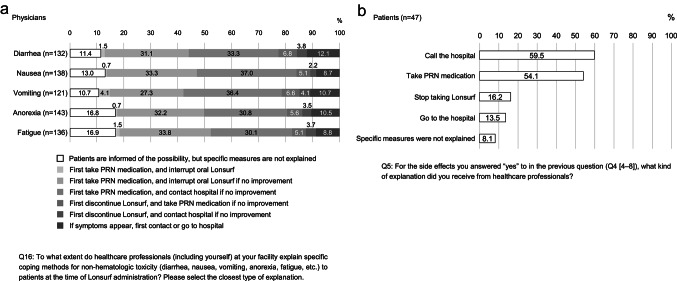
Symptom management instructions. (**a**) Proportion of physicians who explained each type of non-hematologic toxicity – Questionnaire item Q16. PRN (pro re nata): as-needed medication. (**b**) Proportion of patients who reported receiving an explanation of non-hematologic toxicities – Questionnaire item Q5.

Treatment discontinuation due to FTD/TPI-related side effects was most commonly reported in the range of 1–20% (42.8%), followed by 21–40% (28.4%), and 0% (12.6%) (Supplementary Fig. S4). The primary reasons for dose reductions or temporary interruptions were "myelosuppression (neutropenia) " at 64.3%, "non-hematologic toxicity" at 25.6%, and “both equally” (10.1%) (Supplementary Fig. S5). The timing for considering dose reduction or interruption for non-hematologic toxicities was predominantly at Grade 2 for all events, with only a few physicians initiating adjustments at Grade 1 (Supplementary Fig. S6). Similarly, for supportive therapy targeting non-hematologic toxicities, Grade 2 was the most common; however, a certain proportion of physicians also considered supportive therapy at Grade 1, particularly for diarrhea, nausea, and vomiting, as opposed to anorexia and fatigue (Supplementary Fig. S6). When advising patients on specific strategies to manage non-hematologic toxicities, physicians most frequently recommended the use of as-needed (pro re nata: PRN) medications, with 60–70% indicating this approach (Fig. 3a).

From the patient perspective, the most frequently received instruction was to "contact by phone immediately" (59.5%), followed by instructions to "take PRN medications" (54.1%) (Fig. 3b). When analyzed based on age, individuals aged ≥ 70 years were less frequently instructed to take PRN medications and more often advised to call or go to the hospital (Supplementary Fig. S7). A substantial proportion of physicians (approximately 10–15%) provided explanations of non-hematologic side effects without offering specific management instructions; similarly, 8.1% of patients reported receiving explanations about such side effects without concrete management guidance (Fig. 3). Physicians commonly prescribed metoclopramide, domperidone, and prochlorperazine maleate for nausea and vomiting; loperamide hydrochloride and albumin tannate for diarrhea; and dexamethasone, anamorelin hydrochloride, and rikkunshito (a Kampo medicine) for anorexia and fatigue. In medical oncology, olanzapine was more frequently prescribed for nausea and vomiting than in other specialties, indicating variations in prescribing patterns across departments (Supplementary Fig. S8).

**Fig. 4:**
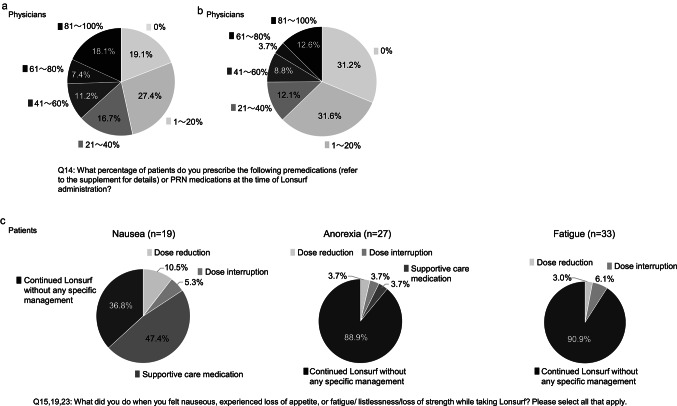
Actual state of supportive care prescriptions and side-effect management. (**a**) Proportion of physicians who prescribed antiemetics as needed (PRN) for patients – Questionnaire item Q14. (**b**) Proportion of physicians who prescribed non-antiemetic medications as needed (PRN) for patients – Questionnaire item Q14. (**c**) Patient-reported management approaches and their frequencies when experiencing nausea, anorexia, or fatigue – Questionnaire item Q15,19,23.

Figure 4 illustrates the proportion of physicians who prescribed medications on a PRN basis. Specifically, 19.1% of physicians did not consider prescribing antiemetic medications on a PRN basis, whereas 31.2% reported the same for non-antiemetic drugs. In contrast, 42.6% of patients reported that they were not prescribed antiemetics (data not shown). Among patients experiencing nausea, the predominant coping strategy was the use of PRN medication (47.4%), and eight of nine reported symptom improvement (data not shown). Conversely, most patients with anorexia and fatigue continued FTD/TPI treatment without adopting specific coping strategies (88.9% and 90.9%, respectively) (Fig. 4c).

**Fig. 5:**
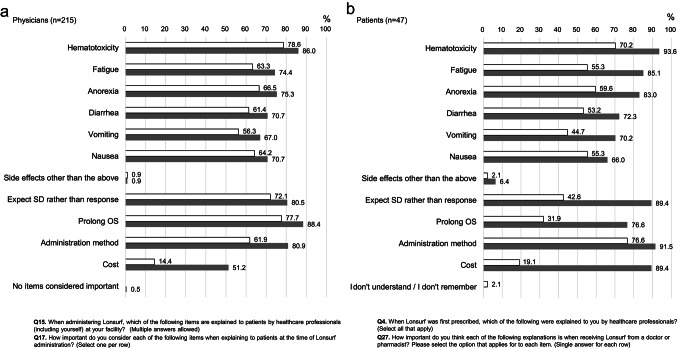
Content and perceived importance of explanations provided at the time of FTD/TPI prescription. (**a**) Content explained to patients at the time of FTD/TPI prescription (white) and the proportion of physicians who considered each item important (“important” + “somewhat important”) (gray) – Questionnaire item Q15,17. (**b**) Content explained by physicians at the time of FTD/TPI prescription (white) and the proportion of patients who considered each item important (“important” + “somewhat important”) (gray) – Questionnaire item Q4,27. OS: Overall Survival, SD: Stable Disease.

At the time of prescription, 72.1% of physicians explained that FTD/TPI “Expect SD rather than response,” and 77.7% indicated that they described it as “a drug that prolongs OS.” In contrast, only 42.6% and 31.9% of patients recalled receiving these explanations. For safety-related information, 78.6% of physicians reported explaining hematologic toxicities, and 70.2% of patients recalled receiving this information. However, approximately 40% of physicians did not explain non-hematologic toxicities, and a similar proportion of patients reported not receiving such explanations (the proportions of physicians and patients reporting explanations about non-hematologic toxicities were 56.3–66.5% and 44.7–59.6%, respectively). Regarding the importance of explanatory content, 89.4% of patients identified “cost” as a crucial explanation item, whereas only 51.2% of physicians considered it important. In practice, 14.4% of physicians provided an explanation about costs, and 19.1% of patients reported having received such an explanation. Overall, physicians showed a small gap between the items they considered important and the content they explained. In contrast, patients regarded many items as important; however, the proportion who felt they had received explanations was generally low (Fig. 5). Regarding healthcare provider involvement, over 80% of both physicians and patients reported the active involvement of “physicians” and “pharmacists” in patient care (Supplementary Fig. S9).

## Discussion

This study represents the first survey to assess the perspectives of both physicians and patients regarding the non-hematologic toxicity of FTD/TPI. Although myelosuppression is the most widely recognized adverse event of FTD/TPI, our findings indicate that non-hematologic toxicities also present substantial clinical challenges for both physicians and patients. Gaps in the recognition of side effects between physicians and patients have been reported for other anticancer agents, and a similar gap was also observed for FTD/TPI in this study^12^. The estimated proportion of each non-hematologic toxicity, as reported by the physician survey, was consistent with those documented in previous clinical trial data (RECOURSE, TAGS)^2,4^; however, patients experienced these symptoms with greater frequency. For all non-hematologic toxicities, patients tended to develop symptoms earlier than anticipated by physicians. The most common schedule for adverse event monitoring during the initial treatment cycle was biweekly (Supplementary Fig. S10), which may have influenced the observed timing of symptom detection. To facilitate the early detection of adverse events, it may be beneficial for patients to visit the hospital on day 8 of cycle 1. Furthermore, incorporating patient-reported outcome (PRO) assessments into routine monitoring may enhance the detection of emerging symptoms and promote timely supportive interventions, ultimately contributing to better symptom control. Previous randomized controlled trials have demonstrated that integrating PRO-based monitoring into cancer care improves symptom control and overall patient outcomes^13^.

Regarding the management of non-hematologic toxicities, physicians advised patients to initially take PRN medication, whereas patients perceived that their first instruction was to contact the hospital by phone. These findings highlight discrepancies in how management instructions were communicated and received by physicians and patients. Notably, a considerable proportion of patients with anorexia and fatigue continued FTD/TPI treatment without adopting any specific coping strategies (Fig. 4c), suggesting that insufficient explanation or understanding of symptom management may have contributed to these behaviors. Ideally, all patients should be informed about strategies for managing side effects; however, the survey indicated that some patients may not have received any explanation about the side effects. In addition, variations by age group were observed in how patients understood or responded to the provided instructions, although it remains unclear whether these differences stem from patients’ comprehension or from differences in how healthcare professionals communicate information. These results underscore the need for further enhancement of the guidance provided by healthcare professionals to patients regarding medication management. Both physicians and patients recognized the active involvement of pharmacists in patient care. Nevertheless, some patients still felt they had not received sufficient explanations. Strengthening collaboration and coordination among physicians, pharmacists, and nurses may help ensure more consistent communication and support for patients, thereby enhancing the delivery of supportive care.

Nausea and vomiting were manageable with antiemetics, highlighting the importance of using PRN medication. However, the patient survey revealed that 42.6% of patients did not receive PRN antiemetics during the treatment period. Similarly, a proportion of physicians indicated in the survey that they had a policy of not prescribing antiemetics on a PRN basis, providing room for further consideration in this area. Differences in management strategies were also observed across facilities and departments. Adverse event management tended to be more effective in university hospitals, cancer centers, and departments of medical oncology (data not shown). Particularly, medical oncology departments used domperidone less frequently and olanzapine more frequently as an antiemetic than other departments did. Given that the choice of drugs varied by department, it is important to select side-effect treatments tailored to each patient while referencing the prescribing patterns from other facilities and departments. Similar variability in oral anticancer drug management across institutions has been reported internationally, underscoring the need for standardized approaches^14^.

However, clear management strategies for fatigue and anorexia have not been established, and more than 90% of the patients who demonstrated such symptoms continued the treatment without any improvement. Development of management methods for fatigue and anorexia is required.

In this survey, the most frequently reported distressing side effects were fatigue, nausea, and anorexia. Although this may be influenced by the fact that FTD/TPI is used as a salvage-line treatment, these symptoms impair the daily lives of patients . Patients perceive non-hematologic toxicities caused by FTD/TPI more strongly than physicians expect. In addition to these side effects, they place greater importance on explanations about drug administration and costs than physicians realize. In the future, healthcare professionals should collaborate to bridge these gaps in knowledge.

However, our results further suggest that discrepancies between physicians’ expectations and patients’ actual experiences, particularly for fatigue and anorexia, may help explain delayed symptom recognition and suboptimal supportive interventions in routine practice. Addressing these perception gaps may therefore require not only pharmacological management, but also systematic patient education, earlier and more frequent symptom assessment, and enhanced multidisciplinary collaboration among physicians, pharmacists, and nurses. Such approaches could help optimize symptom management and improve the overall treatment experience for patients receiving FTD/TPI.

This study has several limitations. First, it is not a survey comparing the subjective views of patients and physicians regarding the same individual. Physicians responded based on their subjective experiences treating their entire group of patients, while patients answered based on their most recent and personal experiences. Second, we used a questionnaire that has not been formally validated, which limits the interpretation and generalizability of the findings. Third, data on concomitant agents such as bevacizumab were not available; therefore, the reported proportions of adverse events may include cases with and without combination therapy. Fourth, the number of recruited physicians and patients was not matched, which may have influenced the results.

In addition, the web-based and voluntary nature of the survey may have introduced selection bias. Patients who participated may not be fully representative of the overall treated population, as individuals who were older, had poorer performance status, or experienced more severe symptoms might have been less likely to respond. This potential selection bias may limit the generalizability of the patient-reported findings. Furthermore, because the total number of individuals who received or viewed the survey invitation could not be determined, precise response rates could not be calculated, raising the possibility of non-response bias.

Future research should aim to clarify further the underlying factors contributing to these discrepancies and to identify practical measures that can enhance patient–physician understanding and improve supportive care in routine oncology practice.

In the context of FTD/TPI therapy, a discrepancy exists between physicians’ and patients’ perceptions of non-hematologic toxicities and the content of explanations, which may pose challenges in effective symptom management. In particular, symptoms such as fatigue and anorexia appear to be less adequately addressed, highlighting the need for continued efforts to improve management strategies.

## Methods

### Study design

This study employed a cross-sectional, observational design using a web-based questionnaire survey. The survey targeted physicians with experience in prescribing FTD/TPI and patients with advanced gastric or colorectal cancer who were receiving or had received FTD/TPI therapy.

### Recruitment of physicians

Physicians were recruited from Plamed Inc.’s registered physician members. Using registered attribute information, Plamed Inc. randomly selected physicians who potentially met predefined eligibility criteria (specialty, facility type, and prescribing experience with FTD/TPI) and electronically dispatched a URL to access the web-based survey.

### Recruitment of patients

Patients were recruited through community pharmacies operated by Nihon Chouzai Co., Ltd. Pharmacists distributed an information sheet containing a URL or QR code to access the web-based questionnaire (hereafter referred to as the *patient information sheet*) to potential participants. Patients accessed the survey independently using their own devices, reviewed the study information online, and provided informed consent prior to participation.

### Eligibility criteria

Eligible physicians were those practicing in Japan with a primary specialty in gastroenterology, oncology, gastrointestinal surgery, or general surgery; affiliated with a medical facility comprising ≥ 100 beds; and who had prescribed FTD/TPI as the primary physician for at least two patients with advanced colorectal or gastric cancer in the preceding year.

Eligible patient participants were adults diagnosed with gastric or colorectal cancer who had experience with FTD/TPI therapy, had received at least two prescriptions of FTD/TPI, had a most recent prescription within the past six months, and provided informed consent to participate in the study.

### Questionnaire development

The questionnaire was developed specifically for this study to capture real-world experiences and perceptions related to non-hematologic toxicities of FTD/TPI. Several items were informed by existing patient-reported outcome concepts, including elements adapted from PRO-CTCAE (Supplementary Table S11, survey form for details)^11^, whereas other questions were developed de novo based on clinical relevance and expert input.

### Data collection

The physician questionnaire assessed treatment scheduling of FTD/TPI, monitoring practices for adverse events, reasons for dose reduction, treatment interruption or discontinuation, perceived frequency and timing of non-hematologic toxicities, supportive care measures, prescription details, instructions provided to patients, and the involvement of healthcare professionals.

The patient questionnaire evaluated treatment status, experiences of dose reduction or interruption, occurrence, severity, and timing of side effects, perceived distress related to symptoms, coping strategies and their effectiveness, prescription status of supportive medications, information received at the time of prescription, and involvement of healthcare professionals.

### Statistical analysis

Categorical variables were summarized using counts and proportions, whereas continuous variables were described using summary statistics, including mean, standard deviation, median, and range. Subgroup analyses described in this manuscript were conducted descriptively to explore potential differences by specialty, facility type, or patient age. These analyses were exploratory in nature, and no formal hypothesis testing was performed.

## Supplementary Information

Supplementary Information 1.

Supplementary Information 2.

Supplementary Information 3.

Supplementary Information 4.

Supplementary Information 5.

Supplementary Information 6.

Supplementary Information 7.

Supplementary Information 8.

Supplementary Information 9.

Supplementary Information 10.

Supplementary Information 11.

## Data Availability

The data underlying this article cannot be shared publicly because of the sponsor’s disclosure policy but is available from the corresponding author on reasonable request. The sponsor’s policy on data sharing may be found at https://www.taiho.co.jp/en/science/policy/clinical_trial_information_disclosure_policy/
