# Supplementary material for: Web questionnaire survey of physicians and patients on the side effects of trifluridine/tipiracil
Source: Sci Rep. 2026 May 22;16:23366. doi: 10.1038/s41598-026-50912-5 (PMC13408580; doi:10.1038/s41598-026-50912-5)
Supplement: Supplementary file 1 — Supplementary Information 1. [file 41598_2026_50912_MOESM1_ESM.pdf]

1A
Physicians

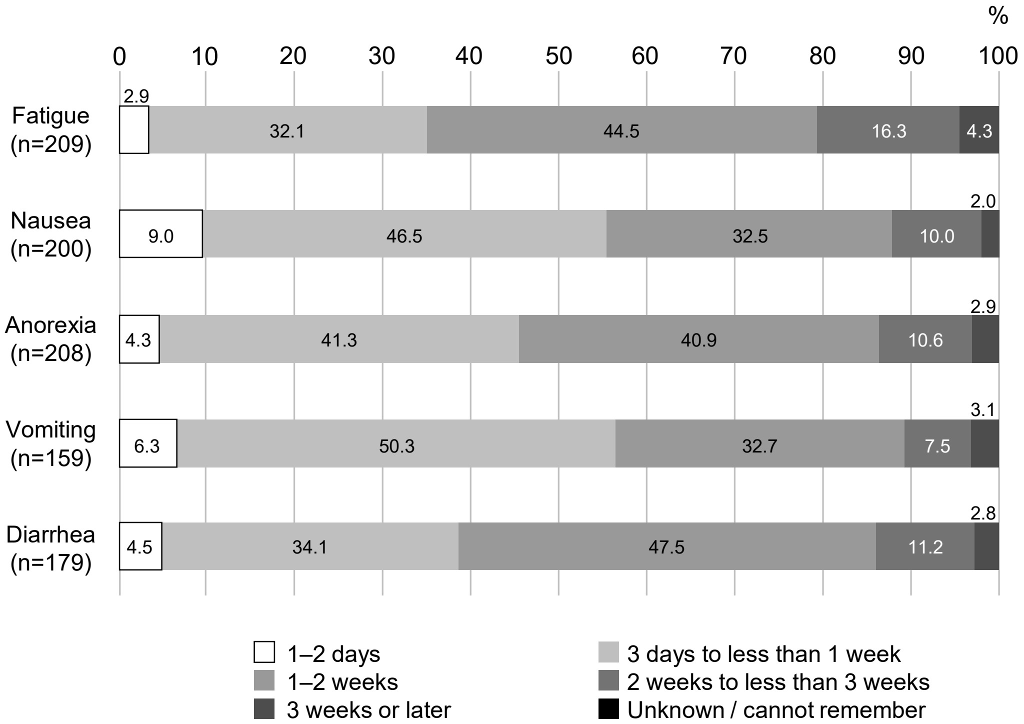

Q8. For each non-hematologic symptom (diarrhea, nausea, vomiting, anorexia,, fatigue), when do these symptoms most commonly occur after starting Lonsurf?

1B
Patients

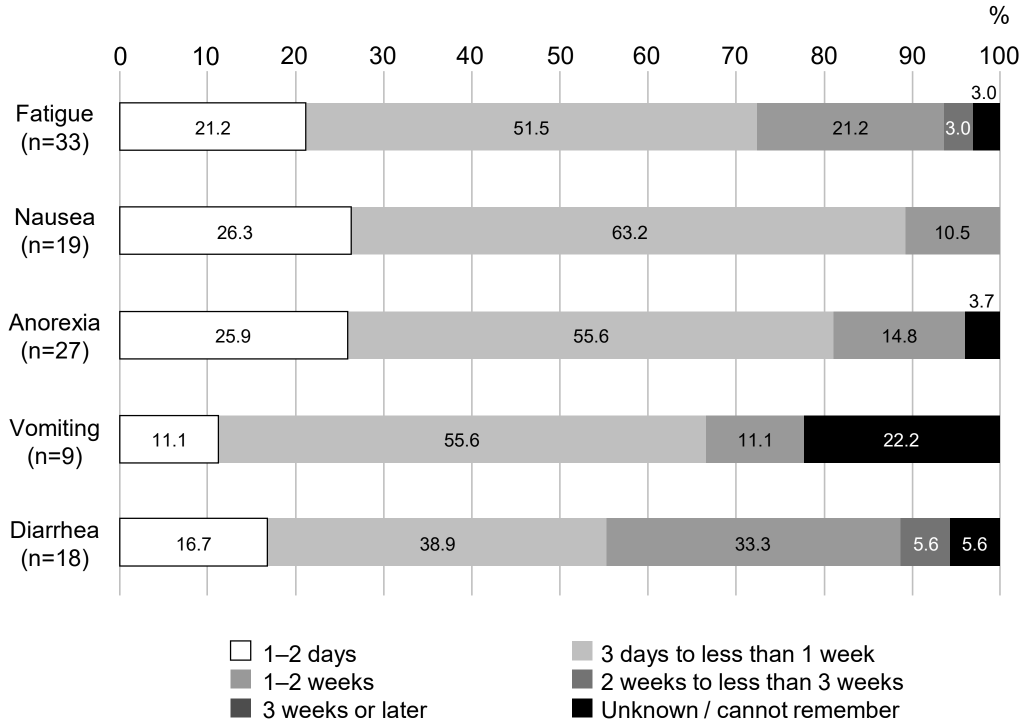

Q10. For each side effect you experienced while taking Lonsurf, how long after starting Lonsurf did the side effect appear? Please select the option that applies to each. Please answer only for side effects that occurred while you were taking Lonsurf.(Single answer for each side effect)

Supplementary Fig. S1 Time to onset of side effects
(1A) Expected time from the date of FTD/TPI prescription to the onset of each adverse reaction (physicians) – Questionnaire item Q8
(1B) Actual time from the date of FTD/TPI prescription to the onset of each adverse reaction (patients) – Questionnaire item Q10
