# Supplementary material for: Web questionnaire survey of physicians and patients on the side effects of trifluridine/tipiracil
Source: Sci Rep. 2026 May 22;16:23366. doi: 10.1038/s41598-026-50912-5 (PMC13408580; doi:10.1038/s41598-026-50912-5)
Supplement: Supplementary file 2 — Supplementary Information 2. [file 41598_2026_50912_MOESM2_ESM.pdf]

2A

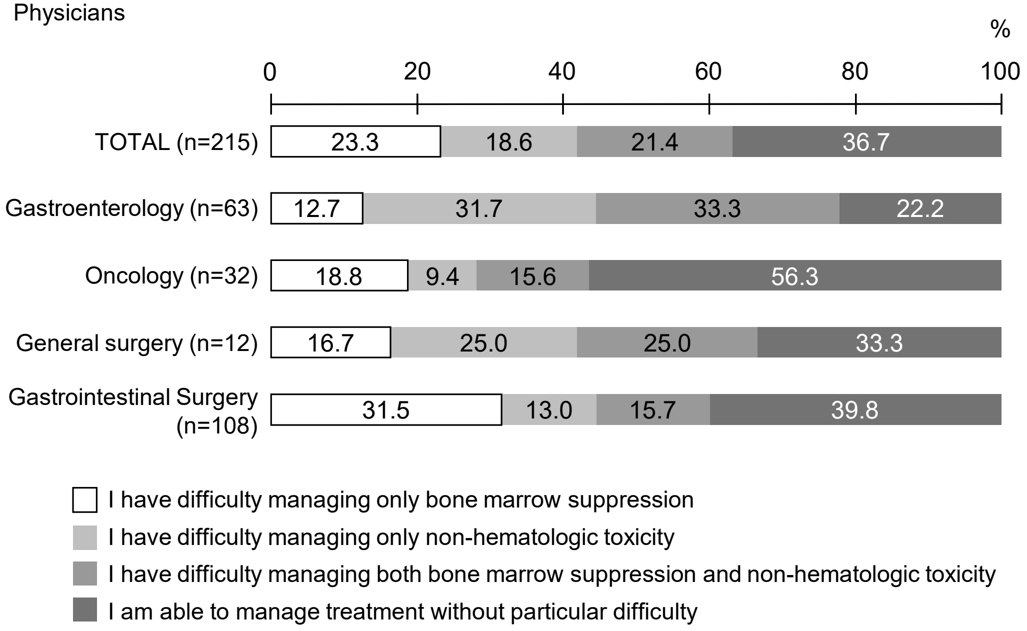

Q6 Please indicate which statement most closely reflects your impression of managing adverse events associated with Lonsurf.

2B

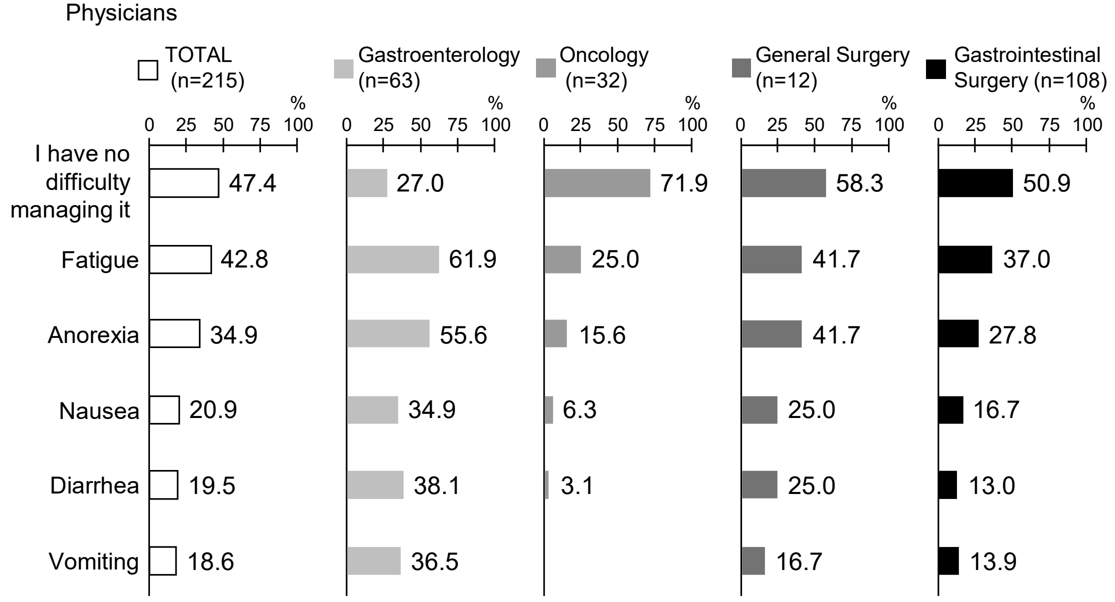

Q13 What is your opinion regarding the management of non-hematologic toxicity (diarrhea, nausea, vomiting, anorexia, fatigue, etc.) in patients receiving Lonsurf?

Supplementary Fig. S2 Perceptions and level of difficulty in managing adverse events by medical specialty (Physicians)
(2A) Perceptions regarding adverse event management by specialty – Questionnaire item Q6
(2B) Level of difficulty in managing each non-hematologic toxicity by specialty (“Having difficulty managing” + “Having some difficulty managing”) – Questionnaire item Q13
