# Supplementary material for: Web questionnaire survey of physicians and patients on the side effects of trifluridine/tipiracil
Source: Sci Rep. 2026 May 22;16:23366. doi: 10.1038/s41598-026-50912-5 (PMC13408580; doi:10.1038/s41598-026-50912-5)
Supplement: Supplementary file 3 — Supplementary Information 3. [file 41598_2026_50912_MOESM3_ESM.pdf]

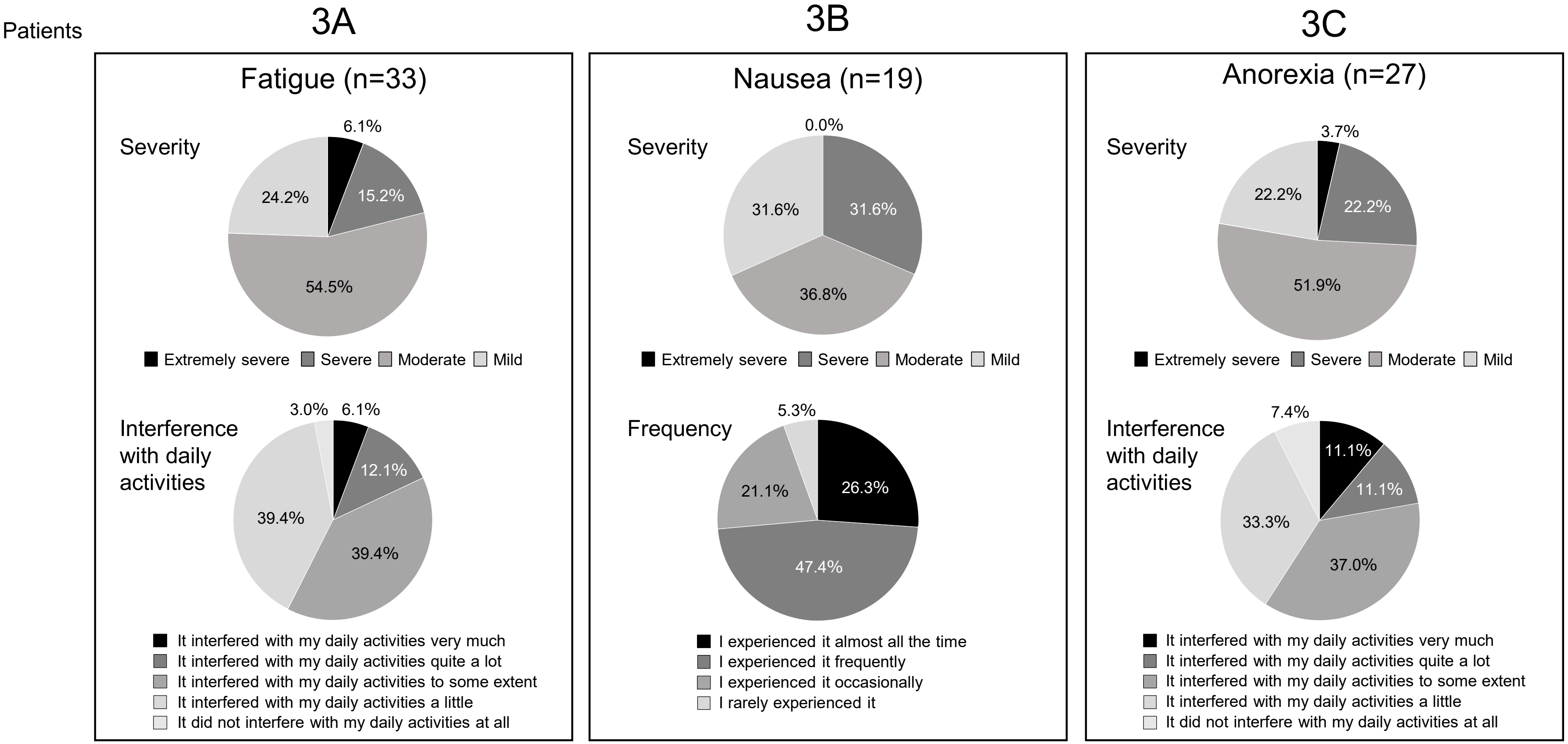

Q21 At its worst, how severe was your fatigue, tiredness, or decreased vitality while taking Lonsurf? Please select the closest option.(Single answer)
Q22 To what extent did fatigue, tiredness, or decreased vitality interfere with your daily life while taking Lonsurf? Please select the closest option.(Single answer)

Q13 How often did you experience nausea (regardless of whether you actually vomited) while taking Lonsurf? Please select the closest option. (Single answer)
Q14 At its worst, how severe was your nausea (regardless of whether you actually vomited) while taking Lonsurf? Please select the closest option.(Single answer)

Q17 At its worst, how severe was your anorexia while taking Lonsurf? Please select the closest option.
Q18 To what extent did anorexia interfere with your daily life while taking Lonsurf? Please select the closest option.(Single answer)

Supplementary Fig. S3 Details of each adverse event (Patients): Based on PRO-CTCAE
(3A) Severity of fatigue and proportion of patients whose daily life was affected – Questionnaire items Q21, Q22
(3B) Severity and frequency of nausea – Questionnaire items Q13, Q14
(3C) Severity of anorexia and proportion of patients whose daily life was affected – Questionnaire items Q17, Q18
