# Supplementary material for: Web questionnaire survey of physicians and patients on the side effects of trifluridine/tipiracil
Source: Sci Rep. 2026 May 22;16:23366. doi: 10.1038/s41598-026-50912-5 (PMC13408580; doi:10.1038/s41598-026-50912-5)
Supplement: Supplementary file 4 — Supplementary Information 4. [file 41598_2026_50912_MOESM4_ESM.pdf]

4A

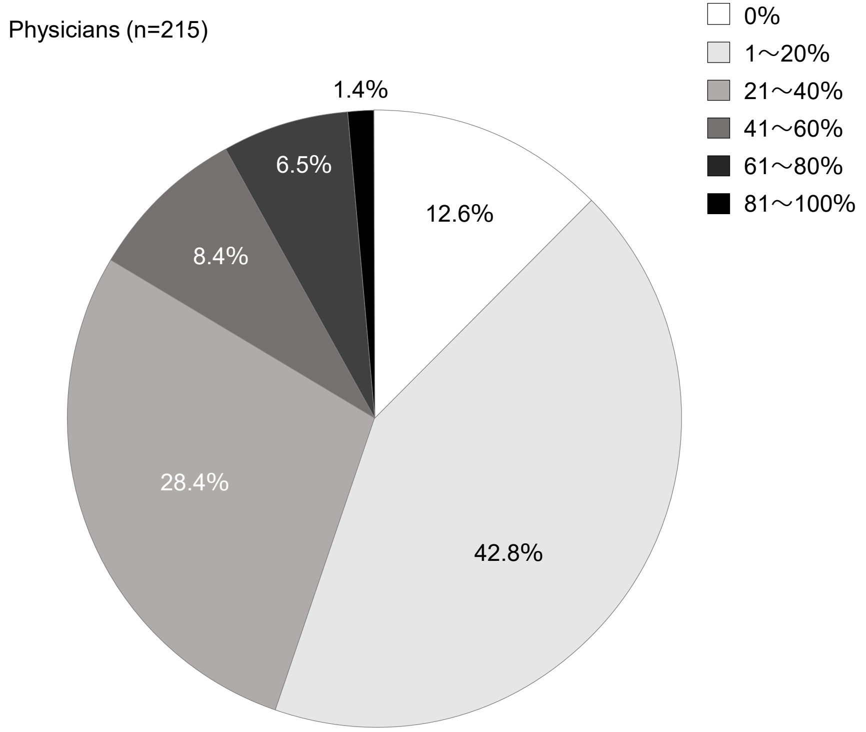

Q4 Based on your experience, what percentage of patients discontinue Lonsurf due to adverse events (not due to disease progression)? Please select the option that most closely applies. (Single answer)

4B

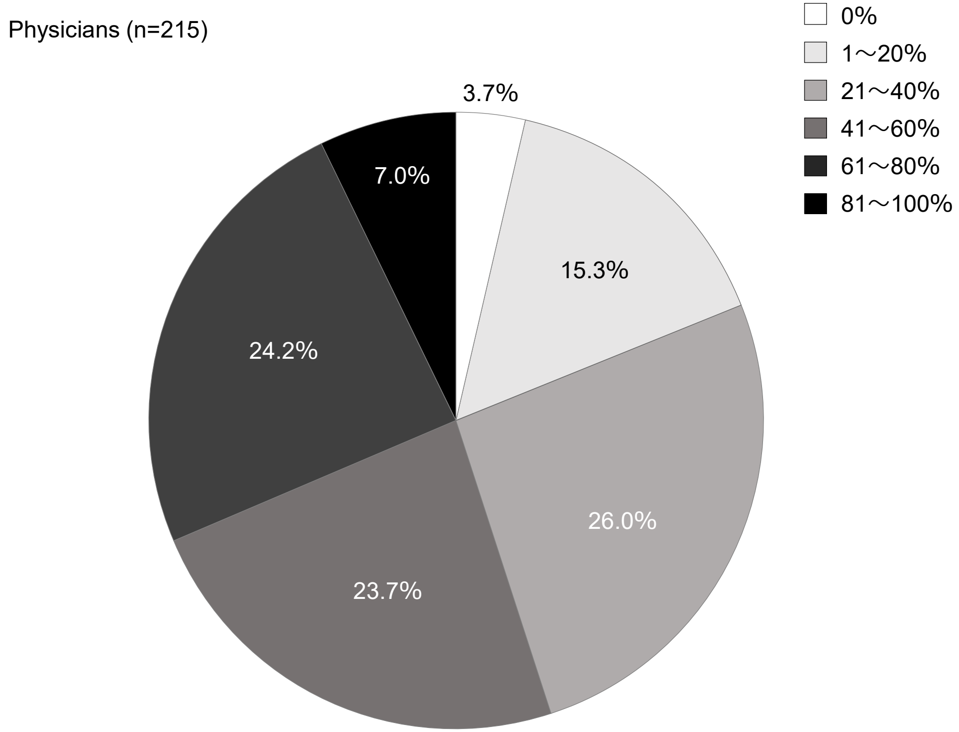

Q3 Based on your experience, what percentage of patients starting Lonsurf require dose reduction or interruption (excluding discontinuation/termination) during administration? Please select the option that most closely applies. (Single answer)

Supplementary Fig. S4 Proportions of FTD/TPI discontinuation and dose reduction/interruption (Physicians, n=215)
(4A) Proportion of FTD/TPI discontinuation due to adverse events – Questionnaire item Q4
(4B) Proportion of FTD/TPI dose reduction or treatment interruption – Questionnaire item Q3
