# Supplementary material for: Web questionnaire survey of physicians and patients on the side effects of trifluridine/tipiracil
Source: Sci Rep. 2026 May 22;16:23366. doi: 10.1038/s41598-026-50912-5 (PMC13408580; doi:10.1038/s41598-026-50912-5)
Supplement: Supplementary file 5 — Supplementary Information 5. [file 41598_2026_50912_MOESM5_ESM.pdf]

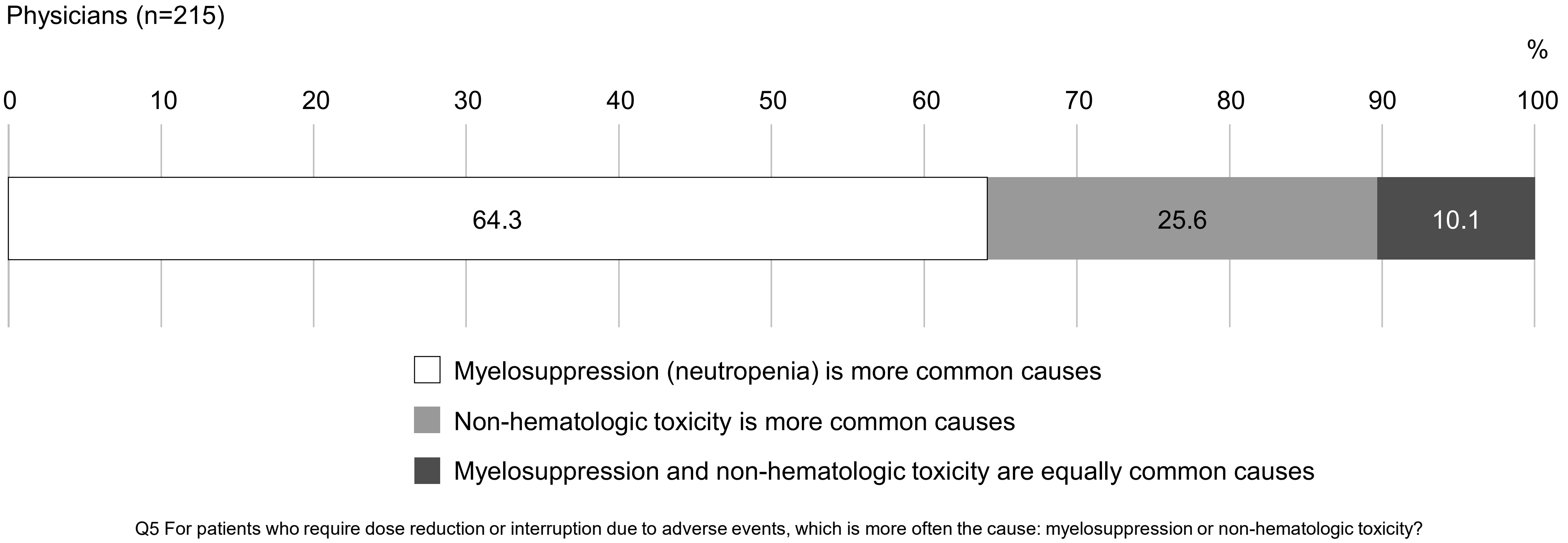

Supplementary Fig. S5 Causes and proportions of FTD/TPI dose reduction or treatment interruption due to adverse events (Physician) – Questionnaire item Q5
