# Supplementary material for: Web questionnaire survey of physicians and patients on the side effects of trifluridine/tipiracil
Source: Sci Rep. 2026 May 22;16:23366. doi: 10.1038/s41598-026-50912-5 (PMC13408580; doi:10.1038/s41598-026-50912-5)
Supplement: Supplementary file 6 — Supplementary Information 6. [file 41598_2026_50912_MOESM6_ESM.pdf]

Physicians (n=215)

		Diarrhea	Nausea	Vomiting	Anorexia	Fatigue
Timing for considering supportive care	Grade1	37.2%	40.5%	46.0%	26.5%	15.3%
	Grade2	55.3%	52.1%	48.8%	60.9%	63.3%
	Grade ≥ 3	6.5%	6.5%	4.7%	8.8%	10.7%
	Supportive care not considered	0.9%	0.9%	0.5%	3.7%	10.7%
Timing for considering dose reduction or treatment interruption	Grade1	9.8%	9.8%	12.6%	11.2%	9.3%
	Grade2	62.8%	60.0%	60.0%	57.2%	54.0%
	Grade ≥ 3	27.4%	30.2%	27.4%	31.6%	36.7%

Q9: For each non-hematologic adverse event, at what grade do you consider supportive therapy or dose reduction/interruption?

Supplementary Fig. S6 Grades at which supportive care, dose reduction, or treatment interruption is considered for each adverse event (physicians)
*Grades are based on CTCAE criteria – Questionnaire items Q9
