# Supplementary material for: Web questionnaire survey of physicians and patients on the side effects of trifluridine/tipiracil
Source: Sci Rep. 2026 May 22;16:23366. doi: 10.1038/s41598-026-50912-5 (PMC13408580; doi:10.1038/s41598-026-50912-5)
Supplement: Supplementary file 7 — Supplementary Information 7. [file 41598_2026_50912_MOESM7_ESM.pdf]

Patients (n=37)

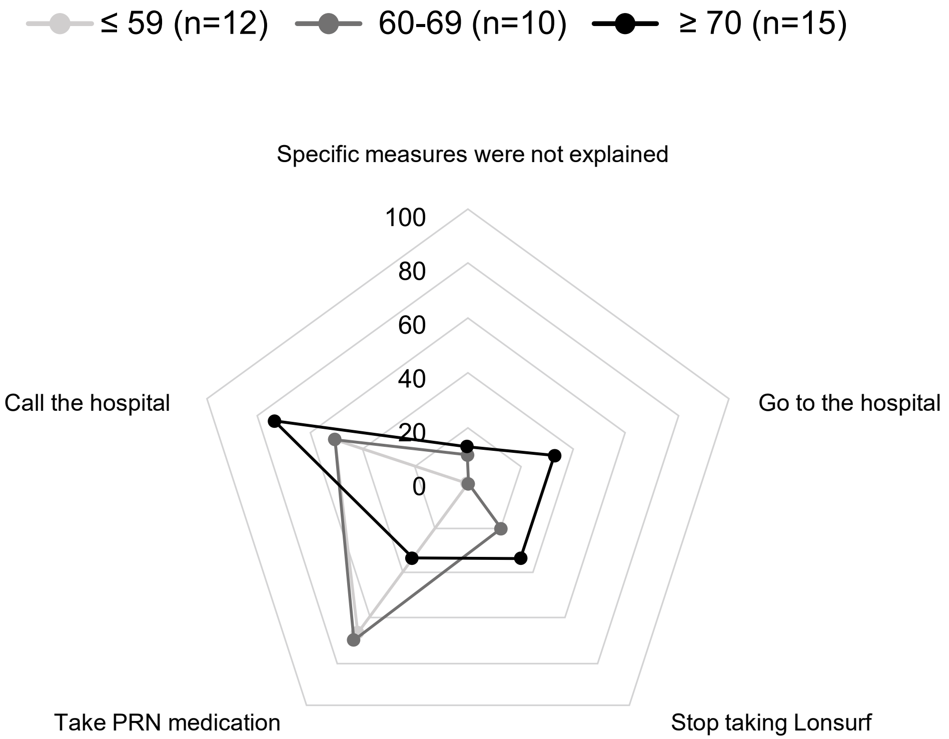

Q5: For the side effects you answered “yes” to in the previous question (Q4 [4–8]), what kind of explanation did you receive from healthcare professionals? (Select all that apply)

Supplementary Fig. S7 Content of explanations patients received regarding the management of nonhematologic toxicities (by age group) – Questionnaire item Q5
