# Supplementary material for: Web questionnaire survey of physicians and patients on the side effects of trifluridine/tipiracil
Source: Sci Rep. 2026 May 22;16:23366. doi: 10.1038/s41598-026-50912-5 (PMC13408580; doi:10.1038/s41598-026-50912-5)
Supplement: Supplementary file 8 — Supplementary Information 8. [file 41598_2026_50912_MOESM8_ESM.pdf]

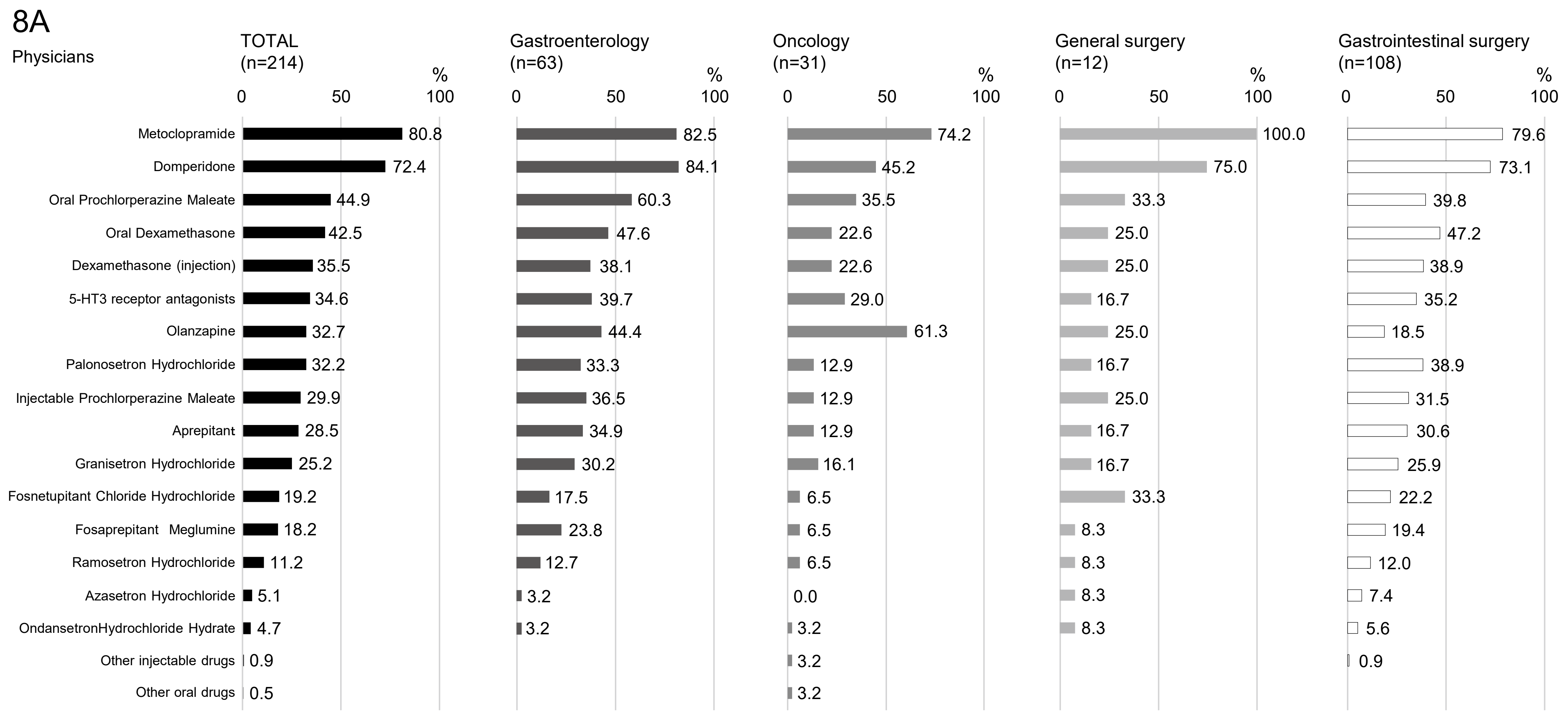

Q11: When providing supportive therapy for "nausea/vomiting" during Lonsurf administration, which medications do you prescribe? (Multiple answers allowed)

Supplementary Fig. S8 Medications prescribed as supportive therapy for FTD/TPI (physicians in gastroenterology, oncology, general surgery, and gastrointestinal surgery)
8A Nausea and vomiting – Questionnaire item Q11

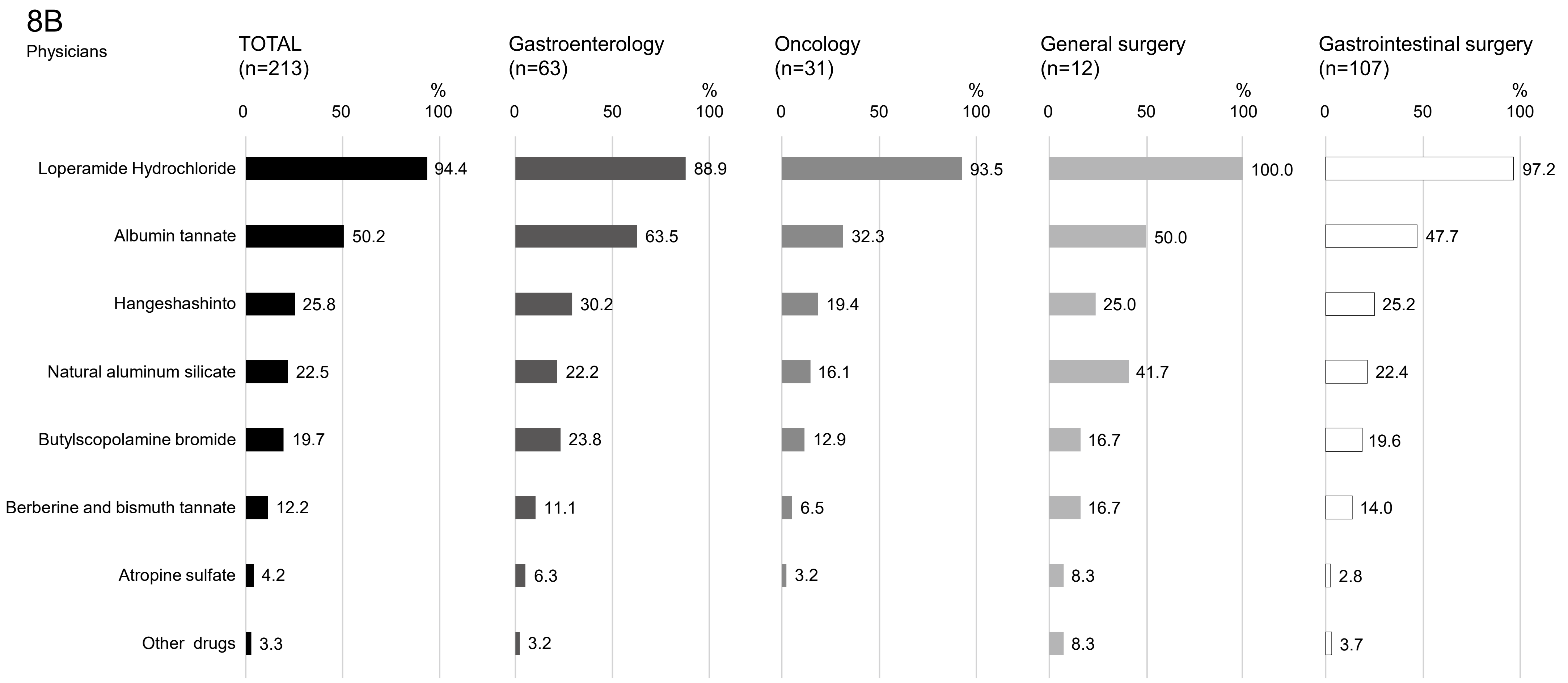

Q10: When providing supportive therapy for "diarrhea" during Lonsurf administration, which medications do you prescribe? (Multiple answers allowed)

8B Diarrhea – Questionnaire item Q10

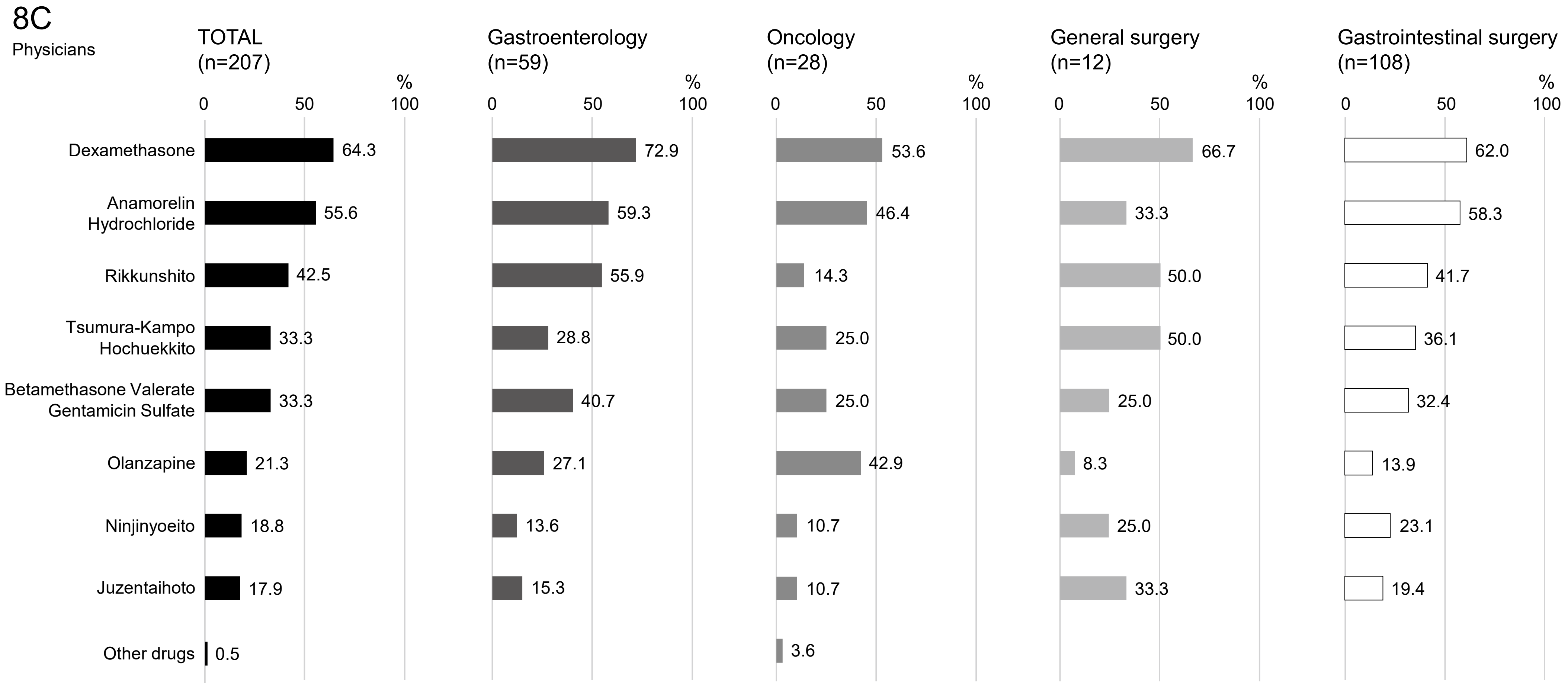

Q12: When providing supportive therapy for "lanorexia/fatigue" during Lonsurf administration, which medications do you prescribe? (Multiple answers allowed)

8C anorexia and fatigue – Questionnaire item Q12
