# Supplementary material for: Web questionnaire survey of physicians and patients on the side effects of trifluridine/tipiracil
Source: Sci Rep. 2026 May 22;16:23366. doi: 10.1038/s41598-026-50912-5 (PMC13408580; doi:10.1038/s41598-026-50912-5)
Supplement: Supplementary file 9 — Supplementary Information 9. [file 41598_2026_50912_MOESM9_ESM.pdf]

9A

Physicians (n=215)

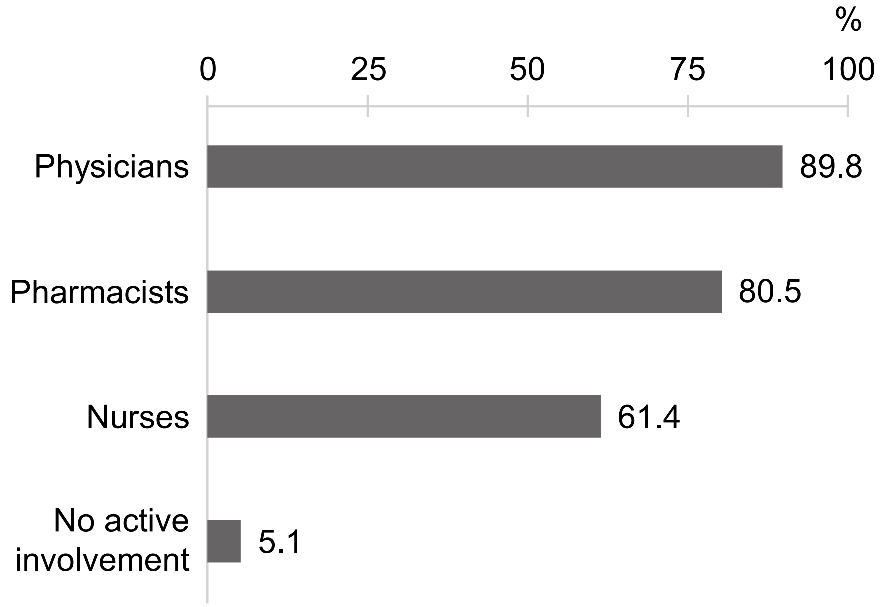

Q18: To what extent are each of the following healthcare professionals involved in patient guidance and follow-up regarding medication adherence for patients prescribed Lonsurf?

9B

Patients (n=47)

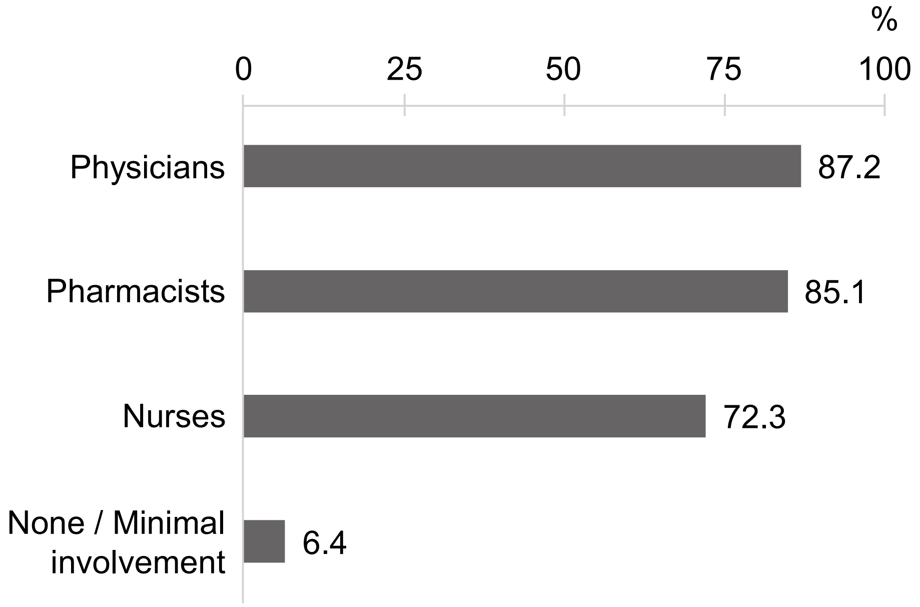

Q25: While taking Lonsurf, to what extent did your doctor or medical staff (pharmacist, nurse) check on your medication status and side effects? Please select the option you think applies.

Supplementary Fig. S9 Degree of healthcare professional involvement (physicians, pharmacists, nurses) in follow-up for patients receiving FTD/TPI therapy
(9A) Physician responses (“Actively involved” + “Somewhat actively involved”) – Questionnaire item Q18
(9B) Patient responses (“Actively checked on medication adherence and side effects and provided appropriate support” + “Regularly checked on medication adherence and side effects”) – Questionnaire item Q25
