# Supplementary material for: Web questionnaire survey of physicians and patients on the side effects of trifluridine/tipiracil
Source: Sci Rep. 2026 May 22;16:23366. doi: 10.1038/s41598-026-50912-5 (PMC13408580; doi:10.1038/s41598-026-50912-5)
Supplement: Supplementary file 10 — Supplementary Information 10. [file 41598_2026_50912_MOESM10_ESM.pdf]

10A

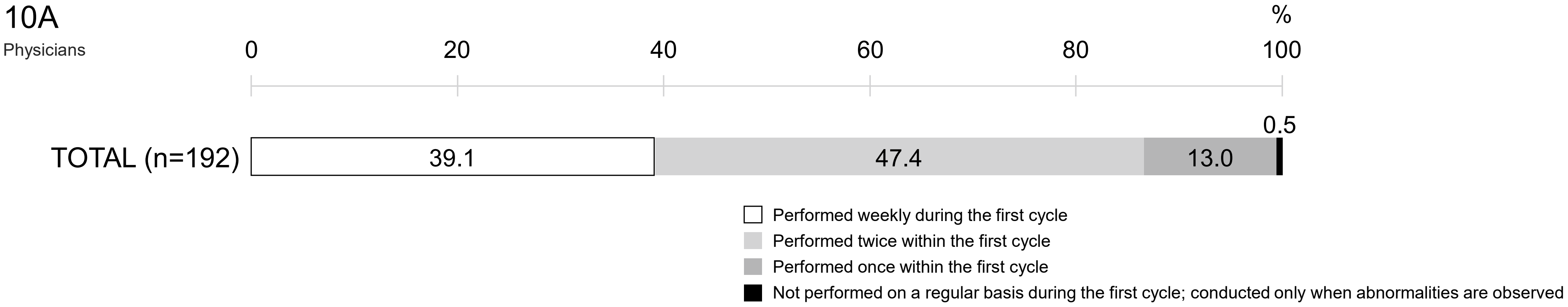

10B

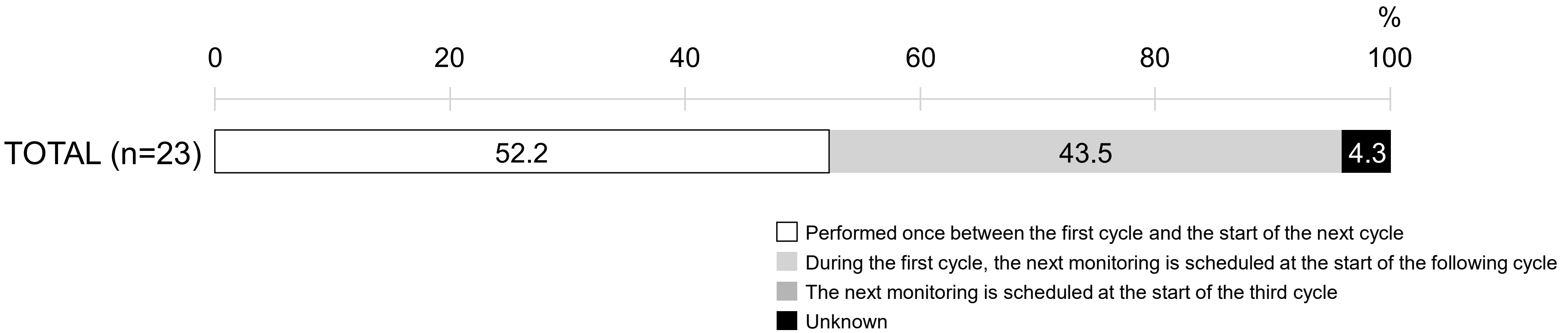

Q2.Regarding adverse event monitoring (regular clinical tests and symptom observation) during the first month of Lonsurf administration, please select the option closest to your current practice.

A. Standard regimen (5 days on, 2 days off ×2, 14 days off: 1 cycle = 28 days)
B. 5 days on, 9 days off (1 cycle = 14 days)

Supplementary Fig. S10 Adverse event monitoring during the first treatment course (physicians)
(10A) Patient monitoring for adverse events during the first cycle of FTD/TPI therapy using the standard regimen (Questionnaire item Q2A)
(10B) Patient monitoring for adverse events during the first cycle of FTD/TPI therapy using the 5 days on, 9 days off schedule (Questionnaire item Q2B)
