# Supplementary material for: Web questionnaire survey of physicians and patients on the side effects of trifluridine/tipiracil
Source: Sci Rep. 2026 May 22;16:23366. doi: 10.1038/s41598-026-50912-5 (PMC13408580; doi:10.1038/s41598-026-50912-5)
Supplement: Supplementary file 11 — Supplementary Information 11. [file 41598_2026_50912_MOESM11_ESM.pdf]

Supplementary Table S11
Questionnaire item（patients）(English Translation)

S0

(Single answer)
If you select "No," the survey will end and you will not receive an honorarium.
1. Yes. I agree to the above and will cooperate with this survey.
2. No. I do not wish to cooperate with this survey.

This survey asks about “Lonsurf” as shown in the image below.
If you have a medication notebook or medication record, please have it ready when answering.

S1
Which cancer did you take Lonsurf for? (Including those currently taking it)
(Single answer)
1. Colorectal cancer
2. Gastric cancer
3. None of the above/Have not taken Lonsurf

If S1=3, end survey. If screened out, a polite message will be displayed.

S2
Please tell us your gender.
(Single answer)
1. Male
2. Female

S3

Please tell us your age.
(Single answer)

1. Under 18 years old (End survey)
2. 18–29 years old
3. 30–39 years old
4. 40–49 years old
5. 50–59 years old
6. 60–69 years old
7. 70–79 years old
8. 80–89 years old
9. 90 years old or older

Q1

Please tell us your current status of taking Lonsurf.
(Single answer)

1. Currently taking, or temporarily stopped but plan/may take again
2. Finished/stopped taking (less than 3 months ago)
3. Finished/stopped taking (3 months to less than 6 months ago)
4. Finished/stopped taking (6 months ago or more)
5. Don’t know / Don’t remember

Q2

For about how long did you take Lonsurf?
(If still taking, please answer the period up to now.)
(Single answer)

1. Less than 1 month
2. 1 to less than 3 months
3. 3 to less than 6 months
4. 6 months to less than 1 year
5. 1 year or more
6. Don’t know / Don’t remember

Q3

When you first started taking Lonsurf, which dosing schedule did you follow? (Single answer)

1. 5 days on, 2 days off, repeated twice, then 14 days off (28-day cycle, see diagram A)
2. 5 days on, 9 days off (14-day cycle, see diagram B)
3. Started with method A, but switched to method B partway through
4. Other dosing schedule
5. Don’t know / Don’t remember

(A)

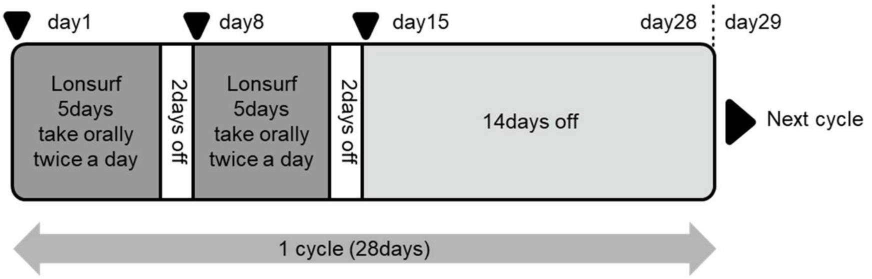

(B)

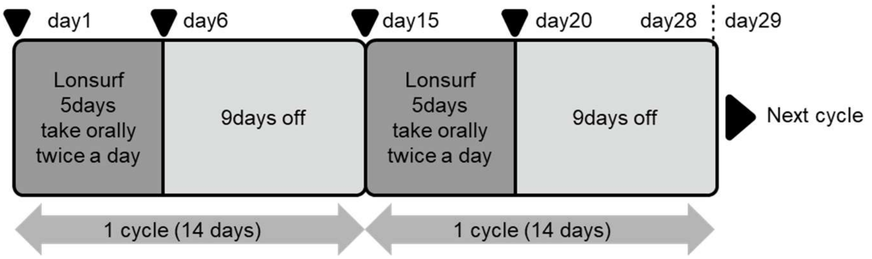

<From here, questions about explanations from healthcare professionals (doctor, pharmacist, nurse, etc.) when Lonsurf was first prescribed.>

Q4

When Lonsurf was first prescribed, which of the following were explained to you by healthcare professionals? (Select all that apply)

1. That it is a drug that prolongs survival
2. That it is not a drug to shrink tumors, but to prevent further growth
3. Side effects detectable by blood tests (decreased white blood cells, neutrophils, platelets, etc.)
4. Diarrhea
5. Nausea
6. Vomiting
7. Anorexia
8. Fatigue, tiredness, decreased vitality
9. Other side effects (please specify, required)
10. Cost
11. How to take Lonsurf
12. Other (please specify, required)
13. Don’t know / Don’t remember (Exclusive)

Q5

For the side effects you answered “yes” to in the previous question (Q4 [4–8]), what kind of explanation did you receive from healthcare professionals? (Select all that apply)

1. Was told there was a possibility of symptoms, but not told specific coping methods (Exclusive)
2. Was instructed to take pre-prescribed PRN (as-needed) medication if symptoms occurred
3. Was instructed to stop taking Lonsurf if symptoms occurred
4. Was instructed to contact by phone immediately if symptoms occurred
5. Was instructed to visit the hospital immediately if symptoms occurred
6. Don’t know / Don’t remember (Exclusive)

Q6

Regarding the prescription and use of antiemetics (medications to suppress nausea/vomiting) while taking Lonsurf:
Which is closest to your experience? (Single answer)

1. Took antiemetics together with Lonsurf
2. Was prescribed antiemetics as needed, but did not take them
3. Was not prescribed antiemetics as needed
4. Don’t know / Don’t remember (Exclusive)

Examples of antiemetics:
Oral: Primperan (metoclopramide), Nauselin (domperidone), Novamin (prochlorperazine), Zyprexa (olanzapine), Decadron (dexamethasone), Emend (aprepitant), 5-HT3 antagonists (ramosetron, granisetron, ondansetron, etc.)

Q7
So far, have you ever reduced the amount of Lonsurf you take at one time, or taken a break from taking it? Please select the option that applies to you.
“Taking a break” refers to stopping Lonsurf for longer than the originally scheduled break, or stopping at an unscheduled time.
(Single answer)

1. No, I always took the prescribed amount on schedule.
2. Reduced the amount at one time during treatment, as instructed by my doctor.
3. Took a break from Lonsurf during treatment, as instructed by my doctor.
4. Both reduced the amount and took a break during treatment, as instructed by my doctor.
5. Reduced the amount or took a break during treatment on my own judgment (without doctor’s instruction).
6. Don’t know / Don’t remember.

Q8
What was the main reason for reducing the amount or taking a break from Lonsurf? Please select the closest option.
“Taking a break” refers to stopping Lonsurf for longer than the originally scheduled

break or stopping at an unscheduled time.
(Single answer)

1. Side effects detectable by blood tests (decreased white blood cells, neutrophils, platelets, etc.)
2. Side effects you could feel yourself (diarrhea, nausea, vomiting, anorexia, fatigue/tiredness/decreased vitality, etc.)
3. Both blood test side effects and self-noticed side effects.
4. Other (please specify, required)
5. Don’t know / Don’t remember.

Q9

Which side effects did you experience while taking Lonsurf? Please select all that apply.

Please answer only for side effects that occurred while you were taking Lonsurf.

(Multiple answers allowed)

1. Side effects detectable by blood tests (decreased white blood cells, neutrophils, platelets, etc.)
2. Diarrhea
3. Nausea (regardless of whether you actually vomited)
4. Vomiting (only if you actually vomited)
5. Anorexia
6. Fatigue, tiredness, decreased vitality
7. Other (please specify, required)
8. Did not experience any side effects while taking Lonsurf
9. Don’t know / Don’t remember

Q10

For each side effect you experienced while taking Lonsurf, how long after starting Lonsurf did the side effect appear? Please select the option that applies to each. Please answer only for side effects that occurred while you were taking Lonsurf.
(Single answer for each side effect)

A. Side effects detectable by blood tests (decreased white blood cells, neutrophils, platelets, etc.)

1. Appeared 1–2 days after starting Lonsurf

2. Appeared 3 days to within 1 week after starting
3. Appeared 1 week to within 2 weeks after starting
4. Appeared 2 weeks to within 3 weeks after starting
5. Appeared after 3 weeks
6. Don’t know / Don’t remember

B. Diarrhea

(Same options as above)

C. Nausea (regardless of whether you actually vomited)

(Same options as above)

D. Vomiting (only if you actually vomited)

(Same options as above)

E. Anorexia

(Same options as above)

F. Fatigue, tiredness, decreased vitality

(Same options as above)

Q11

Of the side effects you experienced while taking Lonsurf, which ones did you find distressing? Please select all that apply.
Please answer only for side effects that occurred while you were taking Lonsurf.
(Multiple answers allowed)

1. Diarrhea
2. Nausea (regardless of whether you actually vomited)
3. Vomiting (only if you actually vomited)
4. Anorexia
5. Fatigue, tiredness, decreased vitality
6. Other (please specify, refer to Q9_7)
7. Did not find any side effect distressing

Q12

Of the side effects you found distressing, which was the most distressing? Please select one from the following.
Please answer only for side effects that occurred while you were taking Lonsurf.
If only one option is displayed, please select it and proceed.

(Single answer)

1. Diarrhea
2. Nausea (regardless of whether you actually vomited)
3. Vomiting (only if you actually vomited)
4. Anorexia
5. Fatigue, tiredness, decreased vitality
6. Other (please specify, refer to Q9_7)

Q13

How often did you experience nausea (regardless of whether you actually vomited) while taking Lonsurf? Please select the closest option.

(Single answer)

1. Hardly ever
2. Occasionally
3. Frequently
4. Almost always
5. Don’t know / Don’t remember

Q14

At its worst, how severe was your nausea (regardless of whether you actually vomited) while taking Lonsurf? Please select the closest option.

(Single answer)

1. Mild
2. Moderate
3. Severe
4. Extremely severe
5. Don’t know / Don’t remember

Q15

When you experienced nausea (regardless of whether you actually vomited) while taking Lonsurf, what did you do? Please select all that apply.

(Multiple answers allowed)

1. Reduced the amount of Lonsurf taken at one time

2. Temporarily stopped taking Lonsurf
3. Took medication to relieve symptoms (if known, please specify the medication name: not required)
4. Continued taking Lonsurf without any particular action
5. Don’t know / Don’t remember

Q16

As a result of your actions in the previous question, did your nausea (regardless of whether you actually vomited) improve? Please select the closest option.
(Single answer)

1. Improved greatly
2. Improved
3. No change
4. Worsened
5. Don’t know / Don’t remember

Q17

At its worst, how severe was your anorexia while taking Lonsurf? Please select the closest option.
(Single answer)

1. Mild
2. Moderate
3. Severe
4. Extremely severe
5. Don’t know / Don’t remember

Q18

To what extent did anorexia interfere with your daily life while taking Lonsurf? Please select the closest option.
(Single answer)

1. Did not interfere at all
2. Interfered a little
3. Interfered to some extent

4. Interfered considerably
5. Interfered very much
6. Don’t know / Don’t remember

Q19

When you experienced anorexia while taking Lonsurf, what did you do? Please select all that apply.

(Multiple answers allowed)

1. Reduced the amount of Lonsurf taken at one time
2. Temporarily stopped taking Lonsurf
3. Took medication to relieve symptoms (if known, please specify the medication name: not required)
4. Continued taking Lonsurf without any particular action
5. Don’t know / Don’t remember

Q20

As a result of your actions in the previous question, did your anorexia improve? Please select the closest option.

(Single answer)

1. Improved greatly
2. Improved
3. No change
4. Worsened
5. Don’t know / Don’t remember

<Questions about fatigue, tiredness, decreased vitality while taking Lonsurf>

Q21

At its worst, how severe was your fatigue, tiredness, or decreased vitality while taking Lonsurf? Please select the closest option.

(Single answer)

1. Mild
2. Moderate

3. Severe
4. Extremely severe
5. Don’t know / Don’t remember

Q22

To what extent did fatigue, tiredness, or decreased vitality interfere with your daily life while taking Lonsurf? Please select the closest option.
(Single answer)

1. Did not interfere at all
2. Interfered a little
3. Interfered to some extent
4. Interfered considerably
5. Interfered very much
6. Don’t know / Don’t remember

Q23

When you experienced fatigue, tiredness, or decreased vitality while taking Lonsurf, what did you do? Please select all that apply.
(Multiple answers allowed)

1. Reduced the amount of Lonsurf taken at one time
2. Temporarily stopped taking Lonsurf
3. Took medication to relieve symptoms (if known, please specify the medication name: not required)
4. Continued taking Lonsurf without any particular action
5. Don’t know / Don’t remember

Q24

As a result of your actions in the previous question, did your fatigue, tiredness, or decreased vitality improve? Please select the closest option.
(Single answer)

1. Improved greatly
2. Improved
3. No change

4. Worsened
5. Don't know / Don't remember

Q25

While taking Lonsurf, to what extent did your doctor or medical staff (pharmacist, nurse) check on your medication status and side effects? Please select the option you think applies.

(Single answer for each row)

	**No check at all (hardly involved)**	**Rarely checked**	**Checked regularly**	**Actively checked and responded appropriately**	**Don't know / Don't remember**
Doctor	1	2	3	4	5
Pharmacist	1	2	3	4	5
Nurse	1	2	3	4	5

Q26

What was the main reason you finally stopped taking Lonsurf? Please select the option that applies.

(Single answer)

1. Stopped due to cancer progression
2. Stopped due to self-noticed side effects (diarrhea, nausea, vomiting, anorexia, fatigue/tiredness/decreased vitality, etc.)
3. Stopped due to side effects detectable by blood tests (decreased white blood cells, neutrophils, platelets, etc.)
4. Stopped due to other reasons (please specify, required)
5. Don't know / Don't remember

<Finally, your opinion on explanations given when Lonsurf is prescribed>

Q27

How important do you think each of the following explanations is when receiving Lonsurf from a doctor or pharmacist? Please select the option that applies to each item.

(Single answer for each row)

Explanation	Not important	Not very important	Neutral	Somewhat important	Important
That it is a drug that prolongs survival	1	2	3	4	5
That it is not a drug to shrink tumors, but to prevent further growth	1	2	3	4	5
Side effects detectable by blood tests (decreased white blood cells, etc.)	1	2	3	4	5
Diarrhea	1	2	3	4	5
Nausea (regardless of whether you actually vomited)	1	2	3	4	5
Vomiting (only if you actually vomited)	1	2	3	4	5
Anorexia	1	2	3	4	5
Fatigue, tiredness, decreased vitality	1	2	3	4	5
Other side effects (please specify: Q13 answer)	1	2	3	4	5
Cost	1	2	3	4	5
How to take Lonsurf	1	2	3	4	5

Questionnaire item（physicians） (English Translation)

S0

(Single answer)

1. Yes. I agree to the above and will cooperate with this survey.
2. No. I do not wish to cooperate with this survey.

<First, we would like to ask about you.>

S1

Please tell us your main department.

(Single answer)

1. Gastroenterology
2. Oncology
3. General Surgery
4. Gastrointestinal Surgery
5. Other department

S2

Please tell us the number of beds at your main facility.

(Single answer)

1. No beds (0 beds)
2. 1–19 beds
3. 20–99 beds
4. 100–199 beds
5. 200–299 beds
6. 300–399 beds
7. 400–499 beds
8. 500 beds or more

S3

Of the patients with unresectable or postoperative recurrent colorectal or gastric

cancer whom you have treated as the primary physician during the past year, please tell us the number of patients to whom you administered Lonsurf in that year.
If there are no applicable patients, please enter 0 (zero).
If you switched from Lonsurf monotherapy to combination therapy, or vice versa, within the past year, please count both cases.
(Enter number)
A. Colorectal Cancer

1. Number of patients administered Lonsurf monotherapy in the past year (0–9999 people/year)
2. Number of patients administered Lonsurf + Avastin (including Bevacizumab BS) in the past year (0–9999 people/year)

B. Gastric Cancer

1. Number of patients administered Lonsurf monotherapy in the past year (0–9999 people/year)
2. Number of patients administered Lonsurf + Cyramza in the past year (0–9999 people/year)

<From here, some questions may include answers without evidence. Please answer based on your own experience and situation.>
<Now, we would like to ask about the usage status of Lonsurf.>
Q1
Regarding the Lonsurf administration schedule (excluding dose reduction or interruption), please select the option closest to your basic policy.
(Single answer)

1. Standard regimen: 5 days on, 2 days off ×2, then 14 days off (28-day cycle)
2. Start with standard regimen for the first cycle, but consider 5 days on, 9 days off, depending on adverse events
3. Use 5 days on, 9 days off regimen from the beginning
4. Other (please specify, required)

Q2
Regarding adverse event monitoring (regular clinical tests and symptom observation) during the first month of Lonsurf administration, please select the

option closest to your current practice.
(Single answer)

A. Standard regimen (5 days on, 2 days off ×2, 14 days off: 1 cycle = 28 days)

1. Conducted weekly during the first cycle
2. Conducted twice during the first cycle
3. Conducted once during the first cycle
4. Not conducted regularly during the first cycle; only when abnormalities are observed

B. 5 days on, 9 days off (1 cycle = 14 days)

1. Conducted once before the next cycle
2. Next monitoring is at the start of the next cycle, even in the first cycle
3. Next monitoring is at the start of the third cycle

Q3

Based on your experience, what percentage of patients starting Lonsurf require dose reduction or interruption (excluding discontinuation/termination) during administration? Please select the option that most closely applies.
(Single answer)

1. 0% (No dose reduction or interruption)
2. 1–20%
3. 21–40%
4. 41–60%
5. 61–80%
6. 81–100%

Q4

Based on your experience, what percentage of patients discontinue Lonsurf due to adverse events (not due to disease progression)? Please select the option that most closely applies.
(Single answer)

1. 0% (No discontinuation due to adverse events; all discontinuations due to progression or other reasons)
2. 1–20%
3. 21–40%
4. 41–60%
5. 61–80%

6. 81–100%

Q5

For patients who require dose reduction or interruption due to adverse events, which is more often the cause: myelosuppression or non-hematologic toxicity?
(Single answer)

1. Myelosuppression (neutropenia) is more common
2. Non-hematologic toxicity (diarrhea, nausea, vomiting, anorexia, fatigue) is more common
3. Both are equally common

Q6

Please indicate which statement most closely reflects your impression of managing adverse events associated with Lonsurf?
(Single answer)

1. Only the management of myelosuppression (neutropenia) presents difficulties
2. Only the management of non-hematologic toxicities (diarrhea, nausea, vomiting, anorexia, fatigue) present difficulties
3. Both the management of myelosuppression and non-hematologic toxicities present difficulties
4. Management is achievable without significant difficulties

Q7

Based on your experience, what is the incidence rate of each symptom among patients receiving Lonsurf (as a percentage of all patients receiving Lonsurf)?
(Select one per row)

**Symptom**	**0% (Never occurred)**	**1–20%**	**21–40%**	**41–60%**	**61–80%**	**81–100%**
Diarrhea	1	2	3	4	5	6
Nausea	1	2	3	4	5	6
Vomiting	1	2	3	4	5	6
Anorexia	1	2	3	4	5	6

Symptom	0% (Never occurred)	1–20%	21–40%	41–60%	61–80%	81–100%
Fatigue (tiredness)	1	2	3	4	5	6

Q8

For each non-hematologic symptom (diarrhea, nausea, vomiting, anorexia, fatigue), when do these symptoms most commonly occur after starting Lonsurf?

(Select one per row)

Symptom	1–2 days after	3 days to <1 week	1–2 weeks	2–3 weeks	After 3 weeks
Diarrhea	1	2	3	4	5
Nausea	1	2	3	4	5
Vomiting	1	2	3	4	5
Anorexia	1	2	3	4	5
Fatigue (tiredness)	1	2	3	4	5

Q9

For each non-hematologic adverse event, at what grade do you consider supportive therapy or dose reduction/interruption?

(Select one per row)

A. Supportive Therapy

Symptom	From Grade 1	From Grade 2	From Grade 3 or higher	Do not consider supportive therapy
Diarrhea	1	2	3	4
Nausea	1	2	3	4
Vomiting	1	2	3	4
Anorexia	1	2	3	4

Symptom	From Grade 1	From Grade 2	From Grade 3 or higher	Do not consider supportive therapy
Fatigue (tiredness)	1	2	3	4

B. Dose Reduction/Interruption

Symptom	From Grade 1	From Grade 2	From Grade 3 or higher
Diarrhea	1	2	3
Nausea	1	2	3
Vomiting	1	2	3
Anorexia	1	2	3
Fatigue (tiredness)	1	2	3

Q10

When providing supportive therapy for "diarrhea" during Lonsurf administration, which medications do you prescribe?

(Multiple answers allowed)

1. Loperamide (loperamide)
2. Tannin albumin (tannin albumin, Tannalbin)
3. Adsorbin (natural aluminum silicate)
4. Feroberin (berberine chloride hydrate, Geranium extract)
5. Atropine
6. Buscopan (butylscopolamine)
7. Hangeshashinto (herbal medicine)
8. Other medications (please specify if selected)

Q11

When providing supportive therapy for "nausea/vomiting" during Lonsurf administration, which medications do you prescribe?

(Multiple answers allowed)

Oral antiemetics:

1. Primperan (metoclopramide)
2. Nauselin (domperidone)
3. Novamin (prochlorperazine)
4. Zyprexa (olanzapine)
5. Decadron (dexamethasone)
6. Emend (aprepitant)
7. 5-HT3 antagonists (e.g., ramosetron, granisetron, ondansetron)
8. Other oral medications (please specify if selected)

Injectable antiemetics: 9. Novamin (prochlorperazine)

10. Kytril (granisetron)
11. Zofran (ondansetron)
12. Aloxi (palonosetron)
13. Seroton (azasetron)
14. Nasea (ramosetron)
15. Proimend (fosaprepitant meglumine)
16. Alokaris (fosnetupitant)
17. Dexart, Decadron (dexamethasone)
18. Other injectable medications (please specify if selected)

Q12

When providing supportive therapy for "anorexia/fatigue" during Lonsurf administration, which medications do you prescribe?
(Multiple answers allowed)

1. Edulmiz (generic: anamorelin)
2. Decadron (generic: dexamethasone)
3. Rinderon (generic: betamethasone)
4. Zyprexa (generic: olanzapine)
5. Rikkunshito (herbal medicine)
6. Juzen-taiho-to (herbal medicine)
7. Ninjin-yoei-to (herbal medicine)
8. Hochu-ekki-to (herbal medicine)
9. Other medications (please specify if selected)

Q13

What is your opinion regarding the management of non-hematologic toxicity (diarrhea, nausea, vomiting, anorexia, fatigue, etc.) in patients receiving Lonsurf?

(Select one per row)

Symptom	Not at all difficult	Not very difficult	Slightly difficult	Difficult
Diarrhea	1	2	3	4
Nausea	1	2	3	4
Vomiting	1	2	3	4
Anorexia	1	2	3	4
Fatigue (tiredness)	1	2	3	4

Q14

What percentage of patients do you prescribe the following premedications or PRN (as-needed) medications at the time of Lonsurf administration?

(Select one per row)

Medication Type	0% (Not prescribed)	1–20%	21–40%	41–60%	61–80%	81–100%
Prophylactic antiemetics at the time of Lonsurf	1	2	3	4	5	6
PRN antiemetics at the time of Lonsurf	1	2	3	4	5	6
PRN medications other than antiemetics at Lonsurf	1	2	3	4	5	6

Q15

When administering Lonsurf, which of the following items are explained to patients by healthcare professionals (including yourself) at your facility?

(Multiple answers allowed)

1. That it is a drug that prolongs survival
2. That it is not a drug to shrink tumors, but to prevent further growth
3. About hematologic toxicity
4. About diarrhea
5. About nausea
6. About vomiting
7. About anorexia
8. About fatigue
9. About other adverse events (please specify if selected)
10. Cost
11. Administration method
12. Other (please specify if selected)

____

____

____

Q16

To what extent do healthcare professionals (including yourself) at your facility explain specific coping methods for non-hematologic toxicity (diarrhea, nausea, vomiting, anorexia, fatigue, etc.) to patients at the time of Lonsurf administration? (Select one per row)

		Only mention possibility	Take PRN medication if symptoms occur			Stop Lonsurf if symptoms occur		Stop Lonsurf and contat hospital
			Stop Lonsurf and contact hospital	If no improvement, stop Lonsurf	If no improvement, contact hospital	icine	If no improvement, contact hospital	
**1**	Diarrhea	1	2	3	4	5	6	7
**2**	Nausea	1	2	3	4	5	6	7

**3**	Vomiting	1	2	3	4	5	6	7
**4**	Anorexia	1	2	3	4	5	6	7
**5**	Fatigue	1	2	3	4	5	6	7

Q17

How important do you consider each of the following items when explaining to patients at the time of Lonsurf administration?

(Select one per row)

**Item**	**Not important**	**Not very important**	**Neutral**	**Somewhat important**	**Important**
That it is a drug that prolongs survival	1	2	3	4	5
That it is not a drug to shrink tumors, but to prevent further growth	1	2	3	4	5
About hematologic toxicity	1	2	3	4	5
About diarrhea	1	2	3	4	5
About nausea	1	2	3	4	5
About vomiting	1	2	3	4	5
About anorexia	1	2	3	4	5
About fatigue	1	2	3	4	5
About other adverse events (refer to Q15_9)	1	2	3	4	5
Cost	1	2	3	4	5
Administration method	1	2	3	4	5

Q18

To what extent are each of the following healthcare professionals involved in patient guidance and follow-up regarding medication adherence for patients prescribed Lonsurf?

(Select one per row)

**Profession**	**Not involved**	**Not very involved**	**Neutral**	**Somewhat involved**	**Actively involved**
Physician	1	2	3	4	5
Pharmacist	1	2	3	4	5
Nurse	1	2	3	4	5

Q19

What is your impression regarding the difference in the frequency of diarrhea, nausea, vomiting, anorexia, and fatigue between colorectal cancer and gastric cancer patients receiving Lonsurf?

(Single answer)

1. Non-hematologic toxicity due to Lonsurf appears more frequently in gastric cancer patients than in colorectal cancer patients
2. Non-hematologic toxicity due to Lonsurf appears more frequently in colorectal cancer patients than in gastric cancer patients
3. The frequency of non-hematologic toxicity is the same in both gastric and colorectal cancer patients
4. I mainly treat either gastric or colorectal cancer patients, so I do not know

<Finally, we would like to ask about you.>

F1

Please tell us the management type of your main facility.

(Single answer)

1. University hospital (national/public/private)
2. National/public hospital (cancer center/specialized cancer hospital)
3. National/public hospital (other than above)

4. General hospital (other corporation, individual, Red Cross, Saiseikai, Koseiren, Kyosai, Kokuho, Kenpo, etc.)

F2

Please select all options applicable to you from the following.
(Multiple answers allowed)

1. Certified oncology drug therapy specialist (certified by the Japanese Society of Medical Oncology [JSMO])
2. Certified cancer treatment physician (certified by the Japanese Board of Cancer Therapy [JBCT])
3. Member of JSMO
4. Member of the Japan Society of Clinical Oncology (JSCO)
5. Main facility is a designated cancer care hospital
6. None of the above
